# Comparison of Goal Scoring Patterns in “The Big Five” European Football Leagues

**DOI:** 10.3389/fpsyg.2020.619304

**Published:** 2021-01-13

**Authors:** Chunhua Li, Yangqing Zhao

**Affiliations:** School of Physical Education and Health, Wenzhou University, Wenzhou, China

**Keywords:** performance analysis, match analysis, player profiles, goal, scoring patterns, European soccer leagues

## Abstract

The objective of the study was to compare goal scoring patterns among the “Big Five” European football leagues during the 2009/2010–2018/2019 seasons. A total of 18 pattern dimensions related to the offense pattern, the shooting situation and the scoring time period were evaluated. Kruskal–Wallis analyses revealed significant pattern differences among the five leagues. The Spanish La Liga showed a greater proportion of goals from throw-ins. The English Premier League had a higher tendency to score from corner kicks. The German Bundesliga had the greatest number of goals from counterattacks and indirect free kicks, and the Italian Serie A had the greatest proportion of penalties. Ligue 1’s scoring ability is weaker than that of the other leagues, especially Bundesliga. The Bundesliga had an overwhelming advantage in goals scored on big chances with assists, while the Premier League had an advantage in goals scored with assists that were not from big chances. However, the differences among the five leagues in the mean goals scored in the last 15 min and the goals from elaborate attacks and direct free kicks were not statistically significant. These results provide a valuable addition to the knowledge of different goal patterns of each league and allow us to better understand the differences among the leagues.

## Introduction

Powered by the so-called “Big Five” football leagues, the English Premier League, the German Bundesliga, the Spanish La Liga, the Italian Serie A, and the French Ligue 1, football has become one of the most profitable areas for the sports and entertainment industry. Europe’s big five leagues are five out of the top seven supported leagues in world football (Poli et al., 2019), and their stellar performances generate enormous television revenues for media groups.

The identification of goal-scoring characteristics and successful attacking strategies is one of the most important components for success in modern elite soccer (Pratas et al., 2018). However, although some studies have partially described how goals are scored in different European leagues (Hughes and Franks, 2005; Armatas et al., 2009; Tenga et al., 2010; Tenga and Sigmundstad, 2011; Wright et al., 2011; Kempe and Memmert, 2018; Zhao and Zhang, 2019), very few studies have compared the differences among top leagues (Mackenzie and Cushion, 2013).

To date, the available studies investigating different leagues have essentially focused on anthropometric measurements (Bloomfield et al., 2005), motor activity performance (Dellal et al., 2011), competitive profile (Vales-Vázquez et al., 2017), competitive balance (Goossens, 2006; Groot, 2008), referee decisions (Prüßner and Siegle, 2015), content analysis (Sarmento et al., 2013), and home advantage (Pollard and Gómez, 2014), while few match analysis studies have been conducted. In particular, Alberti et al. (2013) indicated similar patterns of temporal goal scoring distribution among the English, Italian, Spanish, and French top leagues (2008/09, 2009/10, and 2010/11). Oberstone (2011) compared the English, Italian, and Spanish leagues (2008/09) and found that Serie A was the best passing league with the highest percentage of tackles, while the Premier League had the highest number of tackles and fewer fouls and yellow and red cards. Moreover, La Liga had the highest percentage of shots on target and the highest number of shots converted into goals. However, Yi et al. (2019) suggested Serie A players achieved lower numbers of ball touches, passes and lower pass accuracy per match in UEFA Champions League than players from any of the other four leagues. Konefał et al. (2015) showed that the full-backs from the Spanish La Liga executed the highest number of passes and crosses as well as ball touches in the attacking zone. They also performed the lowest number of passes in the midfield and defensive zones; the highest percentage of passes in these zones was achieved by the full-backs from the German league teams.

Sapp et al. (2018) investigated differences in aggressive play in the top five European football leagues and suggested that the English Premier League is the league with most fouls and cards. After studying 68 matches from four domestic leagues (La Liga, Serie A, Bundesliga, and English Premier League) and Champions League, Sarmento et al. (2018) found differences in the effective offensive sequences between the two. More recently, Mitrotasios et al. (2019) compared goal scoring opportunities in the top four European football leagues. Their result illustrated the significant differences in the four leagues, such as La Liga was good at combination of various offensive methods, English Premier League showed a high degree of direct play, Bundesliga had the most number of counter-attacks, and Italian Serie A showed the shortest offensive sequences.

Since soccer has evolved tactically at the highest level in recent years (Wallace and Norton, 2014; Bradley et al., 2016) and the globalization process has promoted the flow of coaches and players between countries and continents, it would be valuable to investigate the goal scoring characteristics of the top football leagues. Thus, the object of the study was to search for the key similarities and differences among these leagues as well as to find evidence to identify the key factors on the pitch that are associated with a team’s scoring ability within its respective league.

## Materials and Methods

### Samples

Data from the first divisions of the English Premier League (EPL), French Ligue 1 (Ligue 1), German Bundesliga (Bundesliga), Italian Serie A (Serie A), and Spanish La Liga (Liga) were obtained through online sources^1^ with permission. The data resources from Whoscored.com are supported by Opta Sports. Opta debuted its current real-time data collection process for football matches in 2006, leading to an expansion in new data offerings across different sports. Opta data is used in the betting industry, the print and online media, sponsorship, broadcasting, and professional performance analysis. The reliability of the tracking system (OPTA Client System) has been verified by Liu et al. (2013). This study was approved by the local institutional ethics committee.

For the comparisons among leagues, the last nine (2010/11–2018/19 for shooting situations) and ten (2009/10–2018/19 for offense patterns and time periods) seasons were analyzed. Information on the offense pattern, time period, and shooting situation for home and away teams was obtained for each individual match, and both teams were analyzed in each match over the seasons. While the English Premier League, French Ligue 1, Italian Serie A, and Spanish La Liga have 20 teams, the German Bundesliga has 18. Thus, for each season, there were 380 matches used as observations in the first four leagues and 306 for the Bundesliga, meaning we had data for a total of 18,260 matches and 49,483 goals (In the 2013–2014 season of French Ligue 1, victory was awarded to Bastia after Nantes fielded suspended player Abdoulaye Touré. Bastia gained three points, and Nantes’ two goals were cancelled. We still counted the goals from both these teams in the total number of goals). It should be mentioned that we use the average number of goals (different types) scored by a single team in a single season as the sample.

### Variables

The scoresheet records were divided into three main categories. The first category is the time period. The study divided the total time of the game into time periods of 15 min in addition to the time added to the last 15 min at the end of each half (Zhao and Zhang, 2019). The playing time was split into six time periods: 1st–15th min, 16th–30th min, 31st–45th + min, 46th–60th min, 61st–75th min, and 76th–90th + min (hereafter referred to as TP1, TP2, TP3, TP4, TP5, and TP6, respectively).

The second main category is the offense pattern, which is the kind of offense through which the goal was scored. The patterns were divided into eight groups: elaborate attack, counterattack, direct free kick, indirect free kick, corner, throw-in, penalty, and own goal.

The third main category is the goal-shooting situation, which is divided into four groups: goal from a big chance with an assist (Shooting 1), goal with an assist but not from a big chance (Shooting 2), goal from a big chance without an assist (Shooting 3) and goal without both big chance and an assist (Shooting 4). Goal data for the 2009–2010 season were excluded because of a lack of information on big chances and goal assists.

Big Chances: A situation where a player should reasonably be expected to score, usually in a one on one scenario or from very close range when the ball has a clear path to goal and there is low to moderate pressure on the shooter (OPTA, 2019).

Goal Assist: The final touch (pass, pass-cum-shot or any other touch) leading to the recipient of the ball scoring a goal. If the final touch (as defined in bold) is deflected by an opposition player, the initiator is only given a goal assist if the receiving player was likely to receive the ball without the deflection having taken place. Own goals, directly taken free kicks, direct corner goals and penalties do not get an assist awarded (OPTA, 2019).

### Statistical Analysis

Longitudinal data were analyzed using a Kruskal–Wallis *H* test with league as the independent variable. Effect sizes were calculated using the $E_{R}^{2}$ (Tomczak and Tomczak, 2014), which was classified as trivial (<0.01), small (>0.01–0.06), moderate (>0.06–0.14), and large (>0.14), based on guidelines from Kirk (1996). Mann–Whitney *U post hoc* tests were used to compare leagues. To control for type I error, Bonferroni’s correction was applied by dividing the α level by the number of pairwise comparisons being made. Thus, an operational α level of 0.005 (*P* < 0.05/10) was used for league comparisons of each dependent variable.

All analyses were executed in IBM SPSS^®^ Statistics for Windows, version 20.0 (SPSS Inc., Chicago, IL, United States). Offense pattern, shooting situation, and time period data are presented as the mean ± standard deviation (SD).

## Results

### Analysis of the League Effect in Scoring Methods

Between 2009/2010 and 2018/2019, the descriptive results indicate that Bundesliga had the highest mean number of goals from counterattacks (Table 1). This difference in counterattacking is further shown by the very significant league effect (*H* = 136.604, *P* < 0.001, $E_{R}^{2}$ = 0.14). Bundesliga’s mean number of goals from counterattacks was significantly higher than that of the other four leagues [*P* < 0.005; Bundesliga(0.15) > La Liga(0.099) > Ligue 1(0.075) = EPL(0.071); Bundesliga(0.15) > La Liga(0.099) = Serie A(0.09), The mean value in brackets, > means significantly greater than, = means no significant difference]. La Liga’s mean number of goals from counterattacks was significantly higher than those of EPL and Ligue 1. Similarly, there was a significant league effect in the mean number of goals from indirect free kicks in the five leagues (*H* = 29.827, *P* < 0.001, $E_{R}^{2}$ = 0.03), and Bundesliga showed a strong advantage over the other four leagues [*P* < 0.005; Bundesliga(0.113) > La Liga(0.093) = Ligue 1(0.093) = EPL(0.091) = Serie A(0.083)].

**TABLE 1:** Mean frequency values of goals scored by teams according to different offense patterns (*N* = 980).

	EPL *n* = 200	Ligue 1 *n* = 200	Bundesliga *n* = 180	Serie A *n* = 200	La Liga *n* = 200	*H*	*P*	$E_{R}^{2}$
Elaborate attack	0.842 ± 0.338	0.756 ± 0.295	0.834 ± 0.342	0.797 ± 0.29	0.82 ± 0.407	7.913	0.095	0.008
Counter attack	0.071 ± 0.059	0.075 ± 0.06	0.15 ± 0.081^EFIS^	0.09 ± 0.074	0.099 ± 0.082^EF^	136.604	0.000	0.14
Direct free-kick	0.036 ± 0.033	0.039 ± 0.035	0.04 ± 0.04	0.043 ± 0.035	0.039 ± 0.037	8.138	0.087	0.008
Indirect free-kick	0.091 ± 0.052	0.093 ± 0.055	0.113 ± 0.063^EFIS^	0.083 ± 0.049	0.093 ± 0.056	29.827	0.000	0.03
Corner	0.173 ± 0.078^FI^	0.139 ± 0.069	0.161 ± 0.069^F^	0.151 ± 0.069	0.154 ± 0.075	22.556	0.000	0.023
Throw-in	0.014 ± 0.025	0.008 ± 0.017	0.02 ± 0.025^EFI^	0.008 ± 0.016	0.024 ± 0.034^EFI^	68.823	0.000	0.07
Penalty	0.097 ± 0.055	0.108 ± 0.065	0.103 ± 0.06	0.123 ± 0.065^E^	0.11 ± 0.063	17.478	0.002	0.018
Own goal	0.051 ± 0.041^IS^	0.04 ± 0.035	0.035 ± 0.034	0.036 ± 0.032	0.035 ± 0.03	18.558	0.001	0.019

*In the Kruskal–Wallis *H* test, ^*E*^signify that the Bonferroni-adjusted Mann–Whitney *U* test showed a significant difference to English Premier League (*P* < 0.005); ^*F*^signify that the Bonferroni-adjusted Mann–Whitney *U* test showed a significant difference to French Ligue 1 (*P* < 0.005); ^*G*^signify that the Bonferroni-adjusted Mann–Whitney *U* test showed a significant difference to German Bundesliga (*P* < 0.005); ^*I*^showed a significant difference to Italy Serie A (*P* < 0.005); ^*S*^showed a significant difference to Spain La Liga (*P* < 0.005).*

Moreover, a Kruskal–Wallis test to identify differences in goals from throw-ins among the five leagues showed the significant effect of the Bundesliga and La Liga groups (*H* = 68.823, *P* < 0.001, $E_{R}^{2}$ = 0.07). In addition, *post hoc* analysis yielded a significant difference between Bundesliga and La Liga and the other three leagues [La Liga(0.024) = Bundesliga(0.02) > EPL(0.014) = Ligue 1(0.008) = Serie A(0.008)].

However, EPL’s mean number of goals from corners ranks first of all the five leagues (Table 1). A significant league effect was suggested, as shown in Table 1 (*H* = 22.556, *P* < 0.001, $E_{R}^{2}$ = 0.023). EPL scored significantly more corner goals than Ligue 1 or Serie A [*P* < 0.005; EPL (0.173) > Serie A (0.151) = Ligue 1(0.139)].

In terms of own goals, the statistical results are similar to those for corner kicks. EPL had a significantly higher mean number of own goals than La Liga and Serie A [*H* = 18.558, P = 0.001, $E_{R}^{2}$ = 0.019; EPL (0.051) > Serie A (0.036) = La Liga (0.035)].

Finally, an additional *post hoc* analysis showed that there was a significant difference in the number of penalty goals only between Serie A and EPL. It is obvious that more penalty goals took place in Serie A than in EPL [*P* < 0.005; Serie A (0.123) > EPL (0.097)]. There is no significant difference in the mean number of goals from elaborate attacks or from direct free kicks among the five leagues.

### Analysis of the League Effect in Shooting Situations

The comparison of the mean number of goals from the four shooting situations across the five leagues showed significant differences among the leagues (Table 2). In shooting situation 1 (goal from a big chance with an assist), Bundesliga scored significantly more goals than the other four leagues [*H* = 60.611, *P* < 0.001, $E_{R}^{2}$ = 0.069; Bundesliga(0.617) > La Liga(0.527) = EPL(0.468) = Ligue 1(0.465) = Serie A(0.452)].

**TABLE 2:** Mean frequency values of goals scored by teams in different goal-shooting situations (*N* = 882).

	EPL *n* = 180	Ligue 1 *n* = 180	Bundesliga *n* = 162	Serie A *n* = 180	La Liga *n* = 180	*H*	*P*	$E_{R}^{2}$
Shooting 1	0.468 ± 0.214	0.465 ± 0.209	0.617 ± 0.25^EFIS^	0.452 ± 0.191	0.527 ± 0.281	60.611	0.000	0.069
Shooting 2	0.463 ± 0.169^FGS^	0.38 ± 0.127	0.402 ± 0.157	0.437 ± 0.156^F^	0.421 ± 0.181	26.455	0.000	0.03
Shooting 3	0.249 ± 0.098	0.248 ± 0.117	0.271 ± 0.109	0.276 ± 0.105^F^	0.264 ± 0.118	12.510	0.014	0.014
Shooting 4	0.194 ± 0.088^S^	0.172 ± 0.077	0.172 ± 0.073	0.169 ± 0.072	0.162 ± 0.076	14.128	0.007	0.016

*In the Kruskal–Wallis *H* test, ^*E*^signify that the Bonferroni-adjusted Mann–Whitney *U* test showed a significant difference to England Premier League (*P* < 0.005); ^*F*^signify that the Bonferroni-adjusted Mann–Whitney *U* test showed a significant difference to French Ligue 1 (*P* < 0.005); ^*G*^signify that the Bonferroni-adjusted Mann–Whitney *U* test showed a significant difference to German Bundesliga (*P* < 0.005); ^*I*^showed a significant difference to Italy Serie A (*P* < 0.005); ^*S*^showed a significant difference to Spain La Liga (*P* < 0.005).*

In shooting situation 2 (goal with an assist but not from a big chance), the EPL scored significant more goals than Ligue 1, Bundesliga and La Liga (*H* = 26.455, *P* < 0.001, $E_{R}^{2}$ = 0.03), while there was no significant difference in this shooting situation between EPL and Serie A [EPL(0.463) > La Liga(0.421) = Bundesliga(0.402) = Ligue 1(0.38); EPL(0.463) = Serie A(0.432)].

The analysis of the mean number of goals scored in shooting situation 3 (goal from a big chance without an assist) showed a significant league effect in all leagues, in that Ligue 1 scored the fewest goals, and Serie A scored the most goals [*P* < 0.005; Serie A(0.276) > Ligue 1(0.248)].

*Post hoc* analyses showed that EPL scored the most goals of the five leagues from shooting situation 4 (goal without both a big chance and an assist) [*H* = 14.128, *P* = 0.007, $E_{R}^{2}$ = 0.016; EPL (0.194) > Ligue 1(0.162)].

### Analysis of the League Effect in Different Time Periods

Table 3 presents data on the mean number of goals scored in the six identified time periods across the five leagues. From time periods one to five, Bundesliga scored the most goals, while Ligue 1 scored the fewest goals (*P* < 0.005). This suggests that there is a significant difference between Bundesliga and Ligue 1 in the first five time periods. However, a Kruskal-Wallis test revealed that there was no significant difference among all the leagues in time period six (*P* > 0.05).

**TABLE 3:** Mean frequency values of goals scored by teams in different time periods (*N* = 980).

	EPL *n* = 200	Ligue 1 *n* = 200	Bundesliga *n* = 180	Serie A *n* = 200	La Liga *n* = 200	*H*	*P*	$E_{R}^{2}$
TP1	0.169 ± 0.083	0.156 ± 0.08	0.182 ± 0.092^F^	0.168 ± 0.08	0.17 ± 0.088	9.003	0.060	0.009
TP2	0.199 ± 0.091	0.176 ± 0.079	0.217 ± 0.095^F^	0.194 ± 0.085	0.204 ± 0.112	14.994	0.005	0.015
TP3	0.222 ± 0.096	0.198 ± 0.093	0.23 ± 0.101^F^	0.21 ± 0.09	0.219 ± 0.113	11.314	0.023	0.012
TP4	0.24 ± 0.109	0.211 ± 0.097	0.251 ± 0.107^F^	0.234 ± 0.095	0.233 ± 0.109	11.442	0.022	0.012
TP5	0.235 ± 0.101	0.219 ± 0.098	0.254 ± 0.109^F^	0.231 ± 0.098	0.232 ± 0.123	9.561	0.049	0.01
TP6	0.309 ± 0.13	0.299 ± 0.119	0.323 ± 0.124	0.294 ± 0.107	0.315 ± 0.141	6.742	0.150	0.007
Total	1.375 ± 0.442^F^	1.259 ± 0.401	1.457 ± 0.444^F^	1.331 ± 0.381	1.373 ± 0.537	25.060	0.000	0.026

*In the Kruskal–Wallis *H* test, ^*E*^signify that the Bonferroni-adjusted Mann–Whitney *U* test showed a significant difference to England Premier League (*P* < 0.005); ^*F*^signify that the Bonferroni-adjusted Mann–Whitney *U* test showed a significant difference to French Ligue 1 (*P* < 0.005).*

## Discussion

In this paper, we aimed to compare goal scoring patterns in the top five European football leagues over 10 seasons. After exploring the league effects within eight goal types, four shooting situations and six scoring time periods across five major European soccer leagues, we find that different goal types, shooting situations and scoring time periods show varying degrees of league effects.

Some noticeable differences were observed in offense patterns among the different leagues, suggesting differences in either the playing style or the physical demands of the league.

It should be mentioned that counterattack has become an increasingly important means of offense (Maneiro et al., 2019a). And association football have been experienced an evolutionary trend which focus on the defensive and offensive transition (Sarmento et al., 2017). And Sgrò et al. (2016) pointed out that the winning teams tried to maintain a high percentage of ball possession using more accurate and longer pass sequences while the direct play is preferred by the losing team.

Bundesliga have a significant advantage in counterattack goals compared to the other four leagues. Our results are in line with the study of Mitrotasios et al. (2019), in which the Bundesliga had the greatest number of counterattacks in the top five leagues. Another factor to consider is that the full-backs from the German league executed the highest number of inaccurate passes in the defensive and midfield zones compared to their counterparts in the English Premier League, Spanish La Liga, and Italian Serie A (Konefał et al., 2015). This would encourage their opponents to adopt counterattacking tactics since the players lost ball possession in the defensive and midfield zones.

At the same time, La Liga had more goals from fast breaks than Serie A on average—La Liga scores more (although not significantly more), and they are significantly the best of the three at scoring from counterattacks (La Liga, EPL, and Ligue 1).

Full-backs from the Spanish La Liga performed the most passes, crosses and ball touches in the attacking zone (Konefał et al., 2015). They also executed the lowest number of passes in the midfield and defense zones. Therefore, the Spanish defenders tend to press on the side of the opposition, which leaves the defensive zone empty and improves the success rate of the opponent’s counterattack.

A popular assumption is that Serie A is primarily defensive and uses the counterattack to win games (Oberstone, 2011). Its mottos are “do not concede” and “get ten behind the ball” as soon as a goal is scored (Oberstone, 2011). However, the data suggest that this is not how Serie A teams actually play. In fact, Serie A fell in the middle of the top five European leagues in terms of their counterattacking goals. This finding is in agreement with previous research (Oberstone, 2011).

The EPL has a lower number of penalty goals than the other four leagues on average. A possible explanation might be that the referees in the EPL have become more lenient than those in the other leagues (Oberstone, 2011; Sapp et al., 2018).

In regard to the corner kicks goals, De Baranda and Lopez-Riquelme (2012) have compared successful and unsuccessful teams with specific reference to corner kicks and Maneiro et al. (2019b) have identified significant differences in the male and female model of corner kicks execution.

An interesting result is that more own and corner kick goals were scored by the EPL than by the other leagues. Given the EPL’s reputation as an intense league, these results suggest that English Premier League teams are correctly characterized as direct-play teams (Sarmento et al., 2013). One important factor that can explain the differences in corner-kick goals found in this study is the strong heading ability of EPL defenders (Dellal et al., 2011). Dellal suggested that forwards in the EPL lost a greater percentage of heading duels than their La Liga counterparts. Because corner kicks are the main way for a defender to participate in the offense and placing defenders at the goalposts increases the chances of a successful corner kick occurring (De Baranda and Lopez-Riquelme, 2012), the superb heading ability shown by EPL defenders may increase the number of corner kick goals.

Moreover, the EPL is characterized as the toughest marking and fastest game among the EPL, La Liga, and Serie A (Oberstone, 2011). EPL is assumed to have more own goals than are scored in the more elaborate and skilled playing style of La Liga.

The between-league analysis of the shooting situations revealed that Bundesliga excels at goals from shooting situation 1 (goal from a big chance with an assist). As mentioned before, this advantage should be attributed to the poor performance of the German full-backs. According to Bloomfield’s research (Bloomfield et al., 2005), players in the Bundesliga were significantly taller and heavier than players in the other three leagues (all except Ligue 1). Thus, the lack of agility in the Bundesliga’s full-backs could have led to more goals from situation 1.

The EPL does have the highest number of goals from shooting situations 2 and 4 and has significantly more than La Liga, the Bundesliga, and Ligue 1 with respect to shooting situation 2. The EPL, as the “fanciest” league, has earned its reputation as attacking artists when confronted with a solid defense. EPL’s team composition (such as player nationality, game style, statures) may be different from other leagues, which may explain these differences. As far as we know, this has not been explored. Future work should focus on comparing shooting situations between the English Premier League and the lower professional leagues (i.e., Champions League, League 1, and League 2) to investigate whether there are differences.

The five leagues showed nearly identical time period characteristics. The only difference is that the Bundesliga tends to score more goals than Ligue 1. The differences were evident for the French league, probably due to their lower scoring ability. The higher number of goals per match in the Bundesliga may be due to the lower number of matches per season of the league. According to our estimation, in England, the maximum number of competitive games a Premier League club may have to play in 2019/2020 is 74 (38 EPL, eight FA Cup, six League Cup, and 22 Europa League). This is five more games than in Spain (69), nine more than in Italy (65), eight more than in French (66), and 12 more than in Germany (62). If FA cup replays are counted, the gap between the Premier League and the other four leagues becomes even greater.

Last, some of the most interesting findings are those goal scoring patterns that do not statistically separate the five leagues: (1) the mean goals from elaborate attacks per game, (2) the mean goals from direct free kicks per game, and (3) the mean goals scored in the last 15 min. Our study illustrates the similar patterns in the temporal goal scoring distribution among the English, Italian, Spanish, and French leagues, which supports evidence from previous observations (Alberti et al., 2013).

In conclusion, the observed disparities in scoring patterns among leagues may be further explained by cultural differences. Each league has a unique style of play that is undoubtedly influenced by its country’s physical culture and economic, social, and sporting points of view. Further, football has traditionally been proven to be highly resistant to the commodification of its culture. A limitation is that our data do not tell us anything about these cultural differences. Future studies may explore how these parameters account for differences in goal scoring patterns among leagues.

## Conclusion

In summary, our study showed both similarities and differences in various aspects of goal scoring patterns among five major European soccer leagues. There were no significant differences among the five leagues in terms of mean goals scored in the last 15 min or the goals from elaborate attacks and direct free kicks. The Spanish La Liga was better at scoring through throw-ins, the English Premier League showed a high degree of goals from corner kicks, the German Bundesliga had the greatest number of counter-attacks, the Italian Serie A made a higher number of penalty goals and the French Ligue 1 had a weaker scoring ability. These findings have provided valuable source of information adding to our knowledge of understanding the different scoring patterns of each league.

## Data Availability Statement

The raw data supporting the conclusions of this article will be made available by the authors, without undue reservation.

## Ethics Statement

The studies involving human participants were reviewed and approved by the Ethics Committee of School of Physical Education and Health, Wenzhou University. Written informed consent for participation was not required for this study in accordance with the national legislation and the institutional requirements.

## Author Contributions

CL contributed to the study conception and design. YZ and CL performed the material preparation, data collection, and analysis. YZ wrote the first draft of the manuscript and both authors commented on previous versions of the manuscript. Both authors read and approved the final manuscript.

## Conflict of Interest

The authors declare that the research was conducted in the absence of any commercial or financial relationships that could be construed as a potential conflict of interest.

## Footnotes

1 Whoscored.com
